# Inhibition of retinoic acid receptor β signaling confers glycolytic dependence and sensitization to dichloroacetate in melanoma cells

**DOI:** 10.18632/oncotarget.20476

**Published:** 2017-08-24

**Authors:** Cecilie Abildgaard, Christina Dahl, Ahmad Abdul-Al, Annette Christensen, Per Guldberg

**Affiliations:** ^1^ Danish Cancer Society Research Center, Copenhagen, Denmark

**Keywords:** melanoma, cancer metabolism, retinoic acid receptor β, mitochondrial respiration, dichloroacetate

## Abstract

Dysregulation of metabolism during melanoma progression is tightly associated with the acquisition of genetic and epigenetic alterations in regulators of metabolic pathways. Retinoic acid receptor beta (RARβ) is epigenetically silenced in a large proportion of melanomas, but a link between RARβ and metabolic rewiring of melanoma has not been established. Here, we show that in primary human melanocytes, all-trans retinoic acid (a RARβ agonist) induced growth inhibition accompanied by a decrease in both glycolytic and oxidative metabolism, whereas selective inhibition of RARβ led to an increase in the basal glycolytic rate and increased sensitivity to inhibition of glycolysis. In melanoma cells, inhibition of RARβ promoted lower mitochondrial respiration and higher glycolytic activity, which led to energetic stress and activation of the energy sensor AMP-activated protein kinase. This metabolic shift increased the sensitivity to both glycolytic inhibition and stimulation of mitochondrial metabolism with dichloroacetate, an inhibitor of pyruvate dehydrogenase kinase. In melanoma cells harboring the BRAF^V600E^ mutation, RARβ activation antagonized the effect of the BRAF inhibitor PLX4032 (vemurafenib). Collectively, these data suggest that RARβ signaling is involved in regulating cellular metabolism in melanoma and may provide a potential target in combination treatment strategies.

## INTRODUCTION

Melanoma, the most lethal form of skin cancer, causes 50,000 deaths annually with the incidence continuing to increase worldwide. While primary cutaneous melanoma is curable by surgery, the most advanced form of the disease (stage IV) is associated with a 10-year survival of 10-15% [1], reflecting its notorious resistance to conventional anti-cancer therapy. Recent therapeutic advances include immune checkpoint inhibitors and therapies targeting oncogenes or downstream effectors of the MAPK pathway (e.g., BRAF and MEK inhibitors). However, the development of acquired drug resistance eventually leads to relapse in the majority of cases [2, 3].

Melanoma develops from melanin-producing cells, called melanocytes, through the acquisition of multiple genomic alterations. The most common melanoma drivers include activating mutations in *BRAF* and *NRAS* and inactivating mutations or deletions in *CDKN2A* (encoding p16^INK4A^ and p14^ARF^), *PTEN* and *TP53* [4]. Recent evidence suggests that a common function shared among some of these genes is to control cellular metabolism [5, 6]. During the progression of melanoma, cellular metabolism is reprogrammed, implying a shift from mitochondrial respiration toward aerobic glycolysis, leading to increased glucose consumption and lactic acid production (the Warburg effect) [7]. Several reports based on *in vitro* and *in vivo* models of melanoma and clinical studies of melanoma patients have demonstrated a link between activating mutations at codon V600 of *BRAF* (most commonly BRAF^V600E^) and aerobic glycolysis [8–10]. At the molecular level, BRAF^V600E^ regulates oxidative phosphorylation by suppressing the master regulator of mitochondrial biogenesis, PGC1α, through inhibition of the microphthalmia-associated transcription factor (MITF). In contrast, BRAF^V600E^ inhibition leads to oxidative addiction through induction of PGC1α and increased mitochondrial respiration [11]. The corresponding decrease in glycolytic activity can be visualized by PET-CT scanning in melanoma patients treated with BRAF inhibitors, showing a reduced uptake of glucose in the tumor tissue [10]. Phase III clinical trials of the BRAF^V600E^ inhibitor vemurafenib (PLX4032) demonstrated improved overall and progression-free survival in patients with metastatic melanoma [12]. Mitochondrial inhibitors have been suggested as useful adjuvants to BRAF-pathway inhibitors to improve the effect or prevent the development of drug resistance [13–15].

In addition to the well-characterized genetic drivers, the melanoma genome contains numerous epigenetic alterations. One of the recurrent epigenetic targets in melanoma is *RARB* encoding retinoic acid receptor beta (RARβ), which is silenced by promoter hypermethylation in 45-70% of cutaneous melanomas [16, 17]. In cells of the melanocytic lineage, RARβ mediates retinoic acid (vitamin A)-induced growth inhibition and melanogenesis, a marker of melanocytic differentiation [18]. We have previously shown that activation of RARβ in melanocytes induces upregulation of p14^ARF^ [17], which guards against mitochondrial dysfunction and oxidative stress [19]. Here we show that human melanocytes respond to RARβ activation by reducing oxidative metabolism, potentially as part of a differentiation response. In melanoma cells, activation of RARβ antagonizes the effect of PLX4032, whereas inhibition of RARβ induces glycolytic dependence and energetic stress, making the cells vulnerable to treatment with the pyruvate dehydrogenase kinase inhibitor dichloroacetate (DCA).

## RESULTS

### RARβ activation reduces the growth and metabolic rate of melanocytes

**Figure 1:**
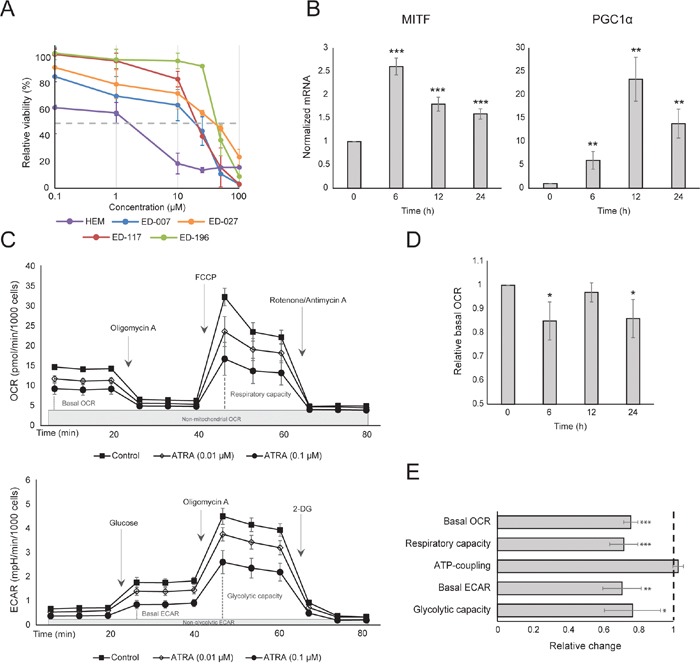
The effect of ATRA on melanocyte metabolism **(A)** Dose-response curves of human epidermal melanocytes (HEM) and melanoma cell lines treated with ATRA (0.1-100 μM) for 6 days. Intersections with the stippled line indicate the IC_50_values. **(B)** Relative expression of MITF and PGC1α mRNA in melanocytes after treatment with ATRA (0.1 μM), normalized to RPLP0 mRNA expression. Three independent experiments were performed on samples representative of multiple (≥3) biological replicates. **(C)** Metabolic profiles of melanocytes treated with two concentrations of ATRA (0.01 and 0.1 μM) for 24 h. The profiles are based on 6 parallel Seahorse XFe96 measurements and are representative of three independent experiments. The top panel depicts the OCR during successive addition of oligomycin A (2 μM), FCCP (1 μM) and rotenone/antimycin (1 μM/1 μM; mito stress test). The lower panel depicts the ECAR during successive addition of glucose (10 mM), oligomycin A (2 μM) and 2-DG (100 mM; glycolytic stress test). **(D)** Relative basal OCR in melanocytes after 6, 12 and 24 h of treatment with ATRA (0.1 μM). **(E)** Summary of the metabolic effects of treatment of melanocytes with ATRA (0.01 μM) for 7 days. The columns indicate the relative shift in basal OCR, respiratory capacity, ATP coupling (relative difference between OCR with and without oligomycin A), basal ECAR and glycolytic capacity based on the mito stress test and the glycolytic stress test. (D and E) Data represent the average ±standard deviation of three biological replicates run in independent experiments. Unpaired t-test was performed to determine statistical significance (^*^p < 0.05; ^**^p < 0.01; ^***^p < 0.001).

We first determined the effect of RARβ activation on the growth of primary human epidermal melanocytes. Cells were treated with the RARβ agonist all-trans retinoic acid (ATRA) for 6 days, and the growth response was determined with a crystal violet-based viability assay. Consistent with previous reports [17, 20, 21], ATRA reduced melanocyte growth in a dose-dependent manner (Figure 1A), with an IC_50_ of 2.4 μM (Table 1). It has been previously shown that short-term treatment (<24 h) with ATRA induces differentiation and melanogenesis in melanocytes, whereas long-term exposure (>24 h) reduces proliferation and induces apoptosis [20, 21]. We found that ATRA (0.1 μM) induced transient up-regulation of the melanocytic lineage-specific transcription factor MITF (microphthalmia-associated transcription factor), with expression peaking after 6 h and then declining towards basal levels (Figure 1B). In melanoma cells, MITF regulates the expression of PGC1α, a marker of an oxidative phenotype [22]. We therefore investigated the expression of PGC1α in melanocytes at different time points after exposure to ATRA (0.1 μM). As shown in Figure 1B, PGC1α was also transiently upregulated, with a ~6-h delay relative to MITF.

**Table 1:** IC_50_ values

Cells/cell lines		Characteristics			IC_50_ values			
**ED number**	**Name**	BRAF status^*^	RARβexpression^**^	**p14^ARF^ expression**	**ATRA (μM)**	**LE135 (μM)**	DCA (mM)^***^	**PLX4032 (μM)**
	HEM#	WT	+	+	2.4±1.6	2.8±0.8	69.1±6.4	NA
ED-007	FM-3	WT	+	-	18.6±8.7	8.6±1.0	12.2±2.2	NA
ED-027	FM-82	BRAF^V600E^	+	+	39.8±5.3	10.7±1.3	17.7±2.1	0.52±0.04
ED-117	Mel-NT3-00	BRAF^V600E^	+	+	25.5±5.0	NA	37.6 ±2.2	0.51±0.09
ED-196	Ma-Mel-51	BRAF^V600E^	+	+	46.2±9.1	8.4±0.4	35.8±3.2	0.26±0.06

IC_50_ values represent mean ± standard deviation of ≥3 independent experiments.

^*^Confirmed by pyrosequencing

^**^Confirmed by qPCR

^***^IC_50_ values published by Abildgaard et al. [29]

#Human epidermal melanocytes

Due to the role of PGC1α in mitochondrial biogenesis, we next investigated whether PGC1α expression correlated with the level of mitochondrial respiration. Using the Seahorse XFe96 instrument, we measured oxygen consumption rate (OCR) and extracellular acidification rate (ECAR), which are indicators of the mitochondrial respiratory rate and glycolytic activity, respectively. OCR and ECAR were measured during the sequential addition of metabolic modulators, enabling the determination of basal rates and capacities of the two energy systems (Figure 1C). To gain insight into the time dependency of ATRA responses, metabolic parameters were measured following both short-term (6-24 h) and long-term (7 days) exposures. Upon treatment of melanocytes with ATRA (0.1 μM) for 6 or 24 h, the basal OCR was reduced. However, after 12 h treatment, OCR was similar to baseline levels (Figure 1D). These fluctuations in the metabolic state coincided with changes in the expression of MITF and PGC1α. Long-term exposure (7 days) to a low dose of ATRA (0.01 μM) resulted in a further decrease in both the basal OCR and respiratory capacity (Figure 1E).

Mitochondrial ATP coupling was unaffected by ATRA (Figure 1E), resulting in a net decrease in ATP production from the mitochondria. PGC1α has been shown to upregulate uncoupling protein 2 (UCP2), leading to a slight mitochondrial uncoupling [23, 24]. UCP2 expression in melanocytes was unaffected by ATRA treatment (Supplementary Figure 1), further supporting that the ATP coupling remained unaltered during RARβ activation. Expression of markers of mitochondrial activity (COX5A, ATP5g1 and NDUFS3) and mitochondrial DNA content were also unaffected by treatment with ATRA (0.1 μM) for up to 24 and 48 h, respectively (Supplementary Figures 1 and 2).

There was no change in the glycolytic rate after 24 h of ATRA treatment (data not shown); however, after 7 days, the basal glycolytic activity and the glycolytic capacity were significantly reduced (Figure 1E). The suppression of both major cellular energy systems indicates that melanocytes exhibit a lower energy demand in the presence of ATRA, which could be a consequence of a reduced cell growth.

### RARβ inhibition increases the basal glycolytic rate and promotes glycolytic dependence in melanocytes

A challenge when studying the cellular effects of ATRA is the presence of unknown concentrations of vitamin A in fetal bovine serum, an essential source of micronutrients in most cell culture media [25]. To study the role of RARβ signaling in melanocyte metabolism in more detail, we therefore used the RARβ antagonist LE135, which targets RARβ with moderate selectivity over RARα and high selectivity over RARγ and RXRα [26].

**Figure 2:**
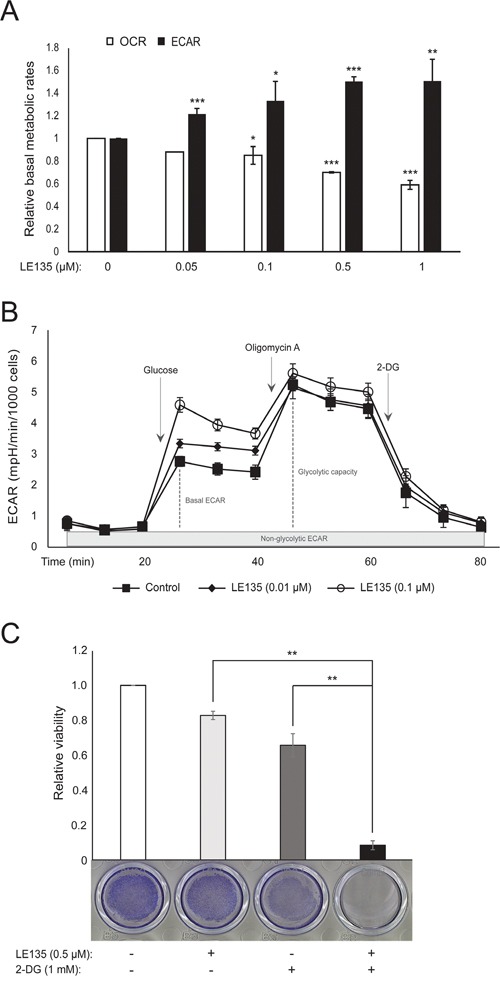
The effect of inhibiting RARβ signaling on melanocyte metabolism **(A)** Basal OCR and basal ECAR of melanocytes after treatment with LE135 at the indicated concentrations for 24 h. **(B)** Changes in basal ECAR and glycolytic capacity after treatment of melanocytes with LE135 (0.01 μM and 0.1 μM) for 7 days. The metabolic profiles are based on the Seahorse XFe96 glycolytic stress test performed on six replicates and are representative of three independent experiments. **(C)** Relative viability of melanocytes after treatment with LE135 (0.5 μM), 2-DG (1 mM) or the combination for 6 days. One-way ANOVA was performed to determine variance between the treatment groups. (A and C) Data represent average values ± standard deviation of three individual experiments. Unpaired t-test (A) and Tukey's HSD test (C) were performed to determine statistical significance (^*^p< 0.05; ^**^p<0.01; ^***^p<0.005).

We repeated the Seahorse protocols shown in Figure 1C on melanocytes treated with different concentrations of LE135 for 24 h and 7 days. After 24 h, we observed a dose-dependent increase in glycolytic activity, with the basal ECAR increasing by up to 50%, and a corresponding reduction in the OCR (Figure 2A). The basal ECAR was still increased after 7 days of treatment (Figure 2B). There was no significant increase in the glycolytic capacity, suggesting that the cells were forced to rely on glycolysis for energy production. This was further supported by a greater sensitivity of these cells to the glycolysis inhibitor 2-deoxy-D-glucose (2-DG), in the presence of LE135 (Figure 2C).

### ATRA antagonizes the effect of BRAF inhibition in melanoma cells

**Figure 3:**
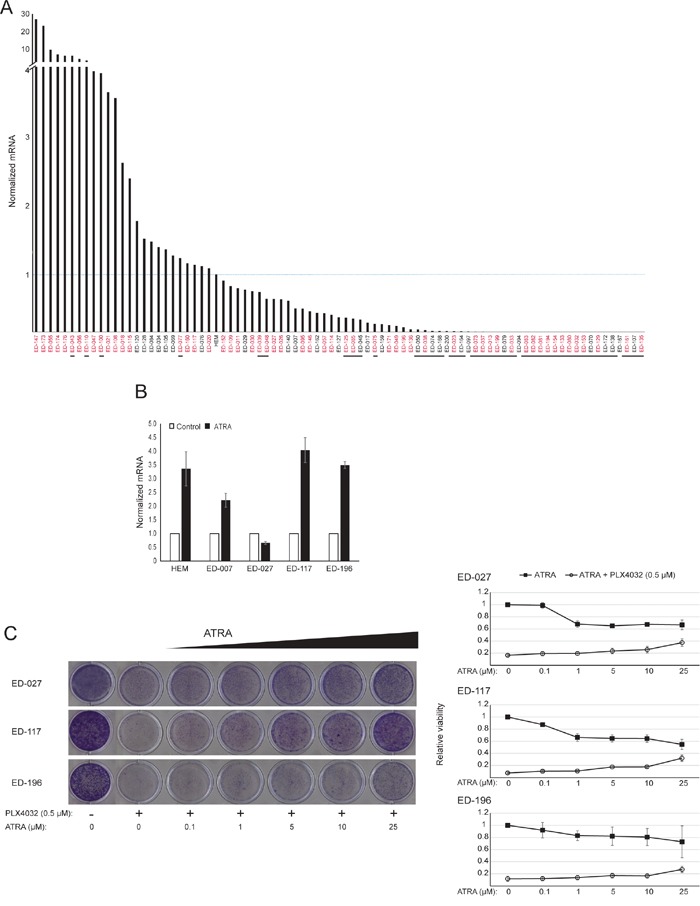
The effect of ATRA on the sensitivity of melanoma cells to BRAF inhibition **(A)** RARβ mRNA expression in 84 human melanoma cell lines relative to melanocytes (HEM). The stippled line indicates the expression in melanocytes. Cell lines with *RARB* promotor hypermethylation are underlined; cell lines with the BRAF^V600E^ mutation are indicated in red. RARβ expression was normalized to RPLP0 expression. Information on *RARB* promotor hypermethylation and *BRAF* status was from Dahl et al. [17]. **(B)** RARβ mRNA expression following treatment with ATRA (1 μM for human epidermal melanocytes [HEM]; 10 μM for melanoma cell lines), normalized to RPLP0 expression. **(C)** Relative viability of three BRAF^V600E^-mutated melanoma cell lines treated for 6 days with PLX4032 (0.5 μM) in combination with different concentrations of ATRA (0.1-25 μM). The left panel displays crystal violet staining representative of three independent experiments. The right panel displays quantification of the relative viability from the absorbance of extracted crystal violet from cells treated with ATRA (0.1-25 μM) in the presence or absence of PLX4032 (0.5 μM). Data represent the average ± standard deviation of three individual experiments.

*RARB* is silenced by promotor hypermethylation in many melanomas, suggesting that it possesses tumor-suppressive properties [16]. In a previous study of genetic and epigenetic events in 110 melanoma cells lines, we found a prevalence of 66% for *BRAF* mutations and 45% for *RARB* promotor hypermethylation, with no correlation between these two events [17]. To extend these data, we examined RARβ expression in 84 of these melanoma cell lines as well as in human epidermal melanocytes. The expression levels varied greatly across melanoma cell lines, ranging from complete lack of expression to levels up to 27 times higher than melanocytes (Figure 3A). As expected, *RARB* promotor hypermethylation was associated with low to undetectable levels of RARβ expression, with few exceptions. There was no association between BRAF^V600E^ mutations and RARβ expression levels (Figure 3A), suggesting that the sensitivity of melanoma cells to ATRA may be independent of *BRAF* status.

To further study the role of RARβ signaling in melanoma, we selected four RARβ-positive melanoma cell lines (ED-007, ED-027, ED-117 and ED-196) for functional analysis. Three of these cell lines were BRAF^V600E^ mutated (ED-027, ED-117 and ED-196) and one was BRAF wild type (ED-007). Treatment with ATRA led to decreased growth of all four cell lines (Figure 1A), although their sensitivity was lower than melanocytes, indicated by the IC_50_ values (Table 1). RARβ expression is known to be induced in response to ATRA [27]. As shown in Figure 3B, RARβ was induced in 3 out of the four melanoma cell lines. Despite a more pronounced RARβ induction in ED-117 and ED-196, the effect of ATRA on MITF and PGC1α expression was attenuated in comparison to melanocytes (Supplementary Figure 3). Suppression of the MITF/PGC1α axis was previously shown to be a consequence of oncogenic BRAF activity [11], which could contribute to a lower response to ATRA. Consistent with this notion, the *BRAF* wild type cells exhibited the highest sensitivity to ATRA, although still considerably lower than melanocytes (Table 1).

To investigate whether targeting BRAF with PLX4032 would restore the sensitivity to ATRA, we treated the BRAF^V600E^-mutant melanoma cell lines with PLX4032 at a concentration close to the IC_50_ values (cf. Table 1) in combination with increasing concentrations of ATRA (0.1-25 μM). Interestingly, ATRA rescued the cytotoxic effect of PLX4032 in all cell lines. The dose-dependent effect of ATRA on the growth of melanoma cells treated with PLX4032 (Figure 3C) indicates antagonism between the two compounds. Treatment with PLX4032 (0.5 μM) did not reduce RARβ expression (Supplementary Figure 4), suggesting a different mechanism for this antagonism.

### The effect of ATRA on cellular metabolism is affected by p14^ARF^ status

**Figure 4:**
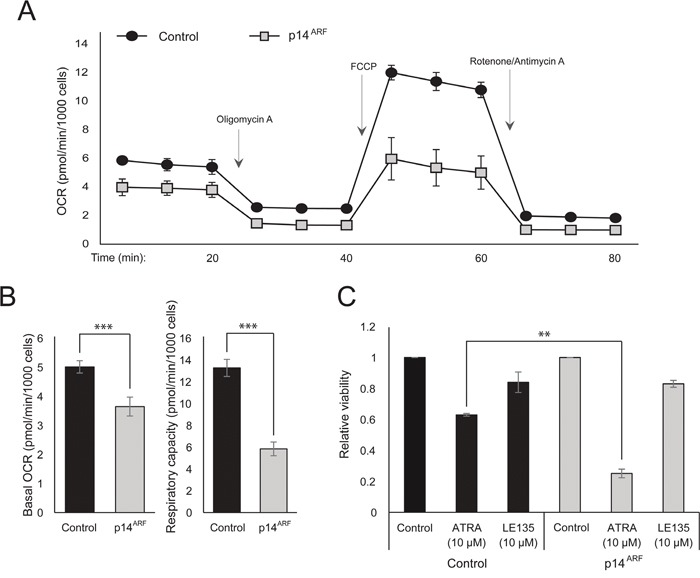
The effect of p14^ARF^ expression on metabolic parameters **(A)** Mitochondrial respiration profiles of ED-007 cells transfected with pEGFP (Control) or pEGFP-p14^ARF^ (p14^ARF^) vectors. OCR measurements were performed according to the Seahorse XFe96 mito stress test including successive addition of oligomycin A (2 μM), FCCP (1 μM) and rotenone/antimycin (1 μM/1 μM). Each measurement is based on six replicates and the profiles are representative of three independent experiments. **(B)** Quantification of the basal OCR and the respiratory capacity (OCR following addition of FCCP) in ED-007 cells transfected with pEGFP (Control) or pEGFP-p14^ARF^ (p14^ARF^) vectors. **(C)** Relative viability of EGFP- and EGFP-p14^ARF^-expressing ED-007 cells after treatment with ATRA (10 μM) or LE135 (10 μM) for 6 days. Data represent the average ± standard deviation of two independent experiments. Unpaired t-test was performed to determine statistical significance (^*^p<0.05; ^**^p<0.01; ^***^p<0.005).

The observation that both ATRA and PLX4032 affect mitochondrial biogenesis could point to a metabolic explanation for the antagonistic effect of these compounds. p14^ARF^ was previously shown to be expressed as a cytoplasmic protein in normal melanocytes and to protect these cells against dysfunctional mitochondria [19]. In accordance with previous findings [17], ATRA increased the expression of p14^ARF^ in RARβ-positive melanoma cells (Supplementary Figure 5). Paradoxically, although p14^ARF^ is frequently lost in melanoma through deletion of the *CDKN2A* locus, it is not possible to stably knock down ARF in melanoma cells expressing this gene ([17]; and data not shown). Instead, to study the potential role of p14^ARF^ in mediating a cellular response to ATRA, we stably transfected the p14^ARF^-deficient ED-007 melanoma cell line with an EGFP-p14^ARF^ construct. The expression of p14^ARF^ in the transfected cells was verified with qPCR (Supplementary Figure 6). Seahorse analysis showed different metabolic profiles (Figure 4A) with a significantly lower basal OCR and respiratory capacity in cells with restored p14^ARF^ expression compared with control-transfected cells (Figure 4B). Interestingly, p14^ARF^-expressing cells also showed an increased sensitivity to ATRA (Figure 4C).

### Blocking RARβ induces glycolytic dependency and energetic stress in melanoma cells and sensitizes them to dichloroacetate

Based on the finding of an antagonistic effect between PLX4032 and ATRA, we tested the combined effect of PLX4032 and LE135 for potential synergism in ED-117 and ED-196 melanoma cells. No cooperative inhibition of melanoma growth was demonstrated in the experimental setup used here (6 days treatment with PLX4032 [0.1 μM] and LE135 [1 μM]; Supplementary Figure 7).

**Figure 5:**
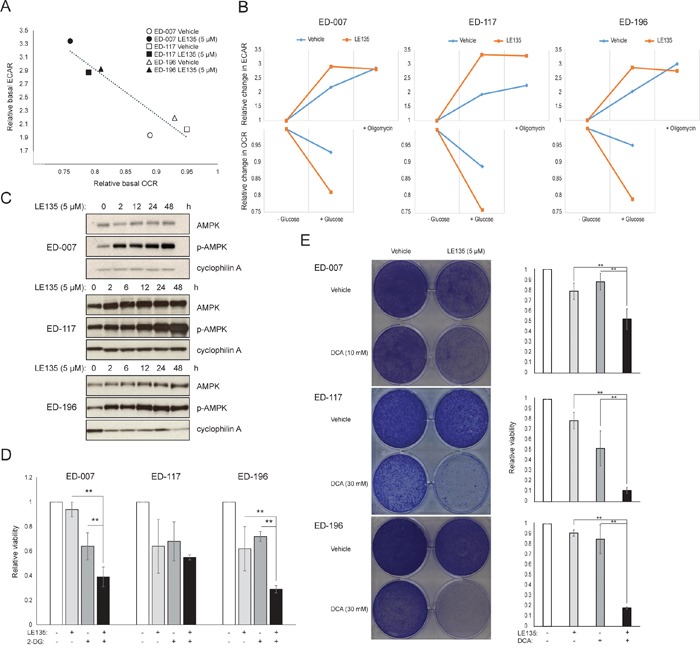
The effect of LE135 on melanoma metabolism **(A)** Graph illustrating the metabolic shift following injection of glucose (10 mM) into the medium of melanoma cell lines treated with LE135 (5 μM) for 24 h compared with untreated controls. Each point indicates the relative changes in OCR and ECAR when glucose is added to the glucose-free medium. **(B)** Relative changes in ECAR (upper panels) and OCR (lower panels) during the injection of glucose, followed by the injection of oligomycin A (only upper panel) for three LE135-treated melanoma cell lines compared to the untreated state. The figure illustrates the shift in the metabolic profile in response to LE135 treatment, with an increased basal glycolytic rate, and the inability to increase the glycolytic rate further when stimulated with oligomycin. **(C)** Immunoblotting of AMPK/p-AMPK after treatment with LE135 (5 μM) for 0-48 h. Cyclophilin A was used as loading control. **(D)** Relative viability of melanoma cell lines after treatment with LE135 (5 μM; except ED-007, 1 μM), 2-DG (1 mM) or the combination for 6 days. **(E)** Viability of melanoma cell lines after treatment with LE135 (5 μM), DCA (10 or 30 mM) or the combination for 6 days. Left panels display crystal violet stainings, right panels display the quantification of the viability relative to the vehicle control. (D and E) Data represent average values ± standard deviation of three independent experiments. One-way ANOVA was performed to determine variance between the treatment groups. Tukey's HSD test was performed to determine statistical significance (^*^p<0.05; ^**^p<0.01; ^***^p<0.005).

To further study the effect of RARβ inhibition on melanoma metabolism, we measured OCR and ECAR in RARβ-positive melanoma cell lines treated with LE135. Similar to what was observed in melanocytes (Figure 2A–2B), LE135 increased the basal glycolytic rate and reduced the basal mitochondrial respiration in melanoma cells (Figure 5A–5B). This metabolic shift, which is consistent with the Warburg effect, was more distinct in melanoma cells compared with melanocytes, leading to an increase in the basal glycolytic rate to reach the maximal capacity. This was illustrated by the inability to further increase ECAR following the addition of oligomycin A, indicating a lack of metabolic flexibility (Figure 5B). To investigate the effect of LE135 on cellular bioenergetics, we investigated the phosphorylation status of AMP-activated protein kinase (AMPK). AMPK senses the cellular energy status by reacting to high levels of AMP, which accumulates as the ATP/ADP ratio drops. Thus, reduced energy production or increased energy consumption can promote a rise in AMP, which binds to AMPK and leads to its phosphorylation and activation [28]. Treatment of melanoma cell lines with LE135 led to phosphorylation of AMPK, indicating that the cells are under energetic stress. The rise in p-AMPK levels was apparent already after 2 h and further increased up to at least 48 h (Figure 5C). These findings were different from those in melanocytes, where treatment with LE135 did not lead to activation of AMPK (Supplementary Figure 8). Both melanoma cells and melanocytes showed a reduction in cell growth during 6 days of treatment with LE135 (IC_50_ values shown in Table 1). Furthermore, similarly to melanocytes, LE135 sensitized melanoma cells to glycolytic inhibition with 2-DG (Figure 5D).

The induction of energetic stress with LE135 in melanoma cells but not in melanocytes alludes to a potential therapeutic relevance of this compound in combination treatment strategies. The pyruvate dehydrogenase kinase inhibitor DCA has previously been shown to inhibit the growth of melanoma cells by inducing a shift in metabolism away from glycolysis, making the cells dependent on mitochondrial respiration [9, 29–32]. Furthermore, DCA has been shown to inhibit the growth of a range of melanoma cell lines, independent of *BRAF* status and PLX4032 sensitivity [29]. To study the combined effect of LE135 and DCA, we applied concentrations lower than the respective IC_50_ values for each of three melanoma cell lines (cf. Table 1). Despite a low effect on growth reduction of each compound individually (9-21% for LE135 and 12-48% for DCA), the combination induced a reduction of up to 89% (Figure 5E). These results suggest that the opposing effects of LE135 (promoting glycolytic dependence) and DCA (shifting the cells away from glycolysis) may act synergistically to inhibit melanoma growth.

## DISCUSSION

ATRA and other vitamin A derivatives reduce cellular growth and induce expression of differentiation markers in various tissues [33, 34]. We found that, in primary human melanocytes, ATRA induces transient upregulation of the MITF/PGC1α axis, consistent with the ATRA-induced increase in mitochondrial function observed in other cell types such as adipocytes and hepatocyes [35–37]. However, long-term treatment with low concentrations of ATRA led to reductions in cellular growth and the metabolic rate, as determined by lower basal glycolysis as well as lower mitochondrial respiration. These metabolic changes in response to ATRA likely reflect a differentiation response towards the non-proliferative state that characterizes melanocytes residing in the skin. Blocking RARβ signaling in these cells resulted in an increase in the basal glycolytic rate and a corresponding decrease in oxidative metabolism. The selective advantage of losing RARβ function in melanoma, for example by *RARB* hypermethylation, could relate to the transition to a more glycolysis-dependent phenotype supporting the Warburg effect.

Contrary to the situation in primary melanocytes, blocking RARβ signaling in melanoma cells led to energetic stress, as indicated by activation of AMPK. This response could be the result of a lower capacity of melanoma cells to increase glycolysis compared with melanocytes. While melanocytes have a relatively low basal glycolytic level and can switch to a higher activity when needed, melanoma cells are characterized by a high glycolytic rate close to their maximal capacity. Thus, melanocytes are more flexible in adapting to inhibition of RARβ signaling to sustain the energy level. This indicates that a relevant therapeutic window exists for LE135 and other RARβ inhibitors in combination therapies, for example with DCA. Similar to the metabolic effects of BRAF inhibition, DCA switches glycolytic cancer cells away from glycolysis towards mitochondrial respiration [9, 38, 39], but unlike PLX4032, the effect of DCA is not limited to BRAF-mutated melanomas [29]. In a previous study, we demonstrated that the metabolic shift induced by DCA correlated with a reduction in ATP levels, suggesting that DCA might target the bioenergetic homeostasis of melanoma cells [29]. Here we found that the combination of LE135 and DCA cooperatively attenuated the growth of melanoma cells expressing the RARβ receptor. Treating melanoma cells with either DCA or LE135 could give them a window to adapt to the new metabolic demands, whereas combining the treatments would limit the metabolic flexibility and make them unable to sustain the energy production required for continued growth.

**Figure 6:**
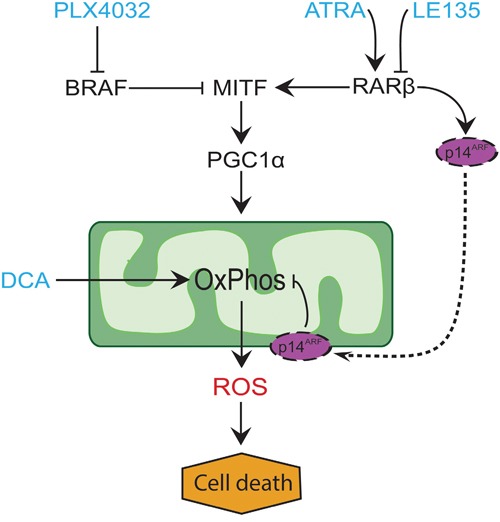
Model illustrating the antagonism between PLX4032 and ATRA Treatment of BRAF^V600E^-mutated melanomas with PLX4032 leads to an upregulation of PGC1α due to reduced suppression of MITF. This increases mitochondrial biogenesis and oxidative phosphorylation (OxPhos) and thereby ROS production, which has a cytotoxic effect on the cells. ATRA has a dual role of both stimulating PGC1α and up-regulating p14^ARF^, the latter serving as a protective mechanism against overproduction of ROS from dysfunctional mitochondria [19]. Thus, ATRA might reduce the sensitivity of RARβ-positive melanomas to PLX4032 through limiting ROS production. DCA also increases OxPhos and ROS production. The combined effect of DCA and LE135 on inhibiting melanoma growth could be due to reduced ATP production, increased ROS production, or both.

In many melanomas, PGC1α expression is low due to suppression of MITF by oncogenic *BRAF* mutations [11]. This phenotype supports the Warburg effect by forcing the cells to switch to glycolysis for energy production. Treatment with BRAF inhibitors restores the expression of PGC1α and shifts the metabolic pattern back towards mitochondrial respiration [11, 40]. The cytotoxic effect of BRAF inhibition may be enhanced due to the presence of dysfunctional mitochondria in melanoma cells, resulting in increased ROS production [13, 41]. We found that ATRA enhances the growth of melanoma cells in the presence of the BRAF inhibitor PLX4032. Based on knowledge from previous studies [11, 17, 19, 41] and the findings presented here, we propose a model integrating the metabolic effects of PLX4032 and ATRA and explaining their antagonism (Figure 6). The model suggests a dual role of increased RARβ signaling, leading to activation of PGC1α and mitochondrial biogenesis, while at the same time suppressing OCR and ROS production through induction of p14^ARF^. The model was supported by findings in the present and an earlier study [17] showing that treatment with ATRA induces the expression of p14^ARF^, and that reconstitution of p14^ARF^-deficient melanoma cells with wild-type p14^ARF^ lowers OCR and increases the sensitivity to ATRA. Thus, ATRA might reduce the sensitivity of RARβ-positive melanomas to PLX4032 by limiting the cytotoxicity from ROS production. Vitamin A has been proposed for prophylactic and therapeutic purposes in many types of cancer, including melanoma [42]. Although clinical validation is lacking, our results argue against the use of vitamin A supplementation in melanoma patients undergoing treatment with BRAF inhibitors, due to the potential antagonizing effect.

In conclusion, we identified a novel function of RARβ signaling in melanocytic and melanoma cell metabolism, which could have clinical implications. The ability of RARβ to activate the MITF-PGC1α pathway, and potentially an p14^ARF^-dependent reduction in mitochondrial respiratory activity, affects the therapeutic response to BRAF inhibition negatively. However, blocking RARβ signaling promotes glycolytic dependence in melanoma cells and enhances the effect of DCA, which could potentially be exploited therapeutically.

## MATERIALS AND METHODS

### Reagents

Sodium dichloroacetate (DCA), 2-deoxy-D-glucose (2-DG), all-trans retinoic acid (ATRA) and LE135 were purchased from Sigma-Aldrich. DCA and 2-DG were dissolved in dH_2_O to a working stock concentration of 1 M. ATRA and LE135 were dissolved in DMSO to a working stock concentration of 0.1 M. PLX4032 (vemurafenib) was purchased from Selleck Chemicals and dissolved in DMSO to a working stock concentration of 0.05 M.

### Cell culture

Melanoma cell lines were obtained from the European Searchable Tumour line Database (ESTDAB, ED) [43]. The status of these cell lines with respect to *BRAF* mutations and *RARB* promoter methylation was described previously [17]. Primary human epidermal melanocytes (neonatal) from lightly pigmented tissue (HEMn-LP; referred to as melanocytes) were purchased from Invitrogen (C0025C). Melanocytes from three different individuals were used for the experiments (Lot no 200706893 from Mar. 2015 and Nov 2016; Lot no 1583282 from Feb. 2017). Melanoma cell lines were cultured at 37°C under 5% CO_2_ in RPMI-1640 medium supplemented with 10% fetal bovine serum. HEMn-LP cells were cultured under the same conditions in 254CF medium supplemented with 1% human melanocyte growth supplement (HMGS) including phorbol 12-myristate 13-acetate (PMA). For experimental setups, HEMn-LP cells were cultured in medium supplemented with HMGS-2 (without PMA). All media and supplements were purchased from Invitrogen.

### Cell viability analysis

A crystal violet assay was applied to evaluate the effect of the studied compounds on cell viability. Cells were seeded in duplicates and treated with the relevant compounds or vehicle control for 6 days. Medium and treatment compounds were replaced every 48 h. Experiments were repeated three times independently. After the treatment period, medium and unattached cells were removed, and the remaining cells were washed in PBS and fixed with glutaraldehyde for 15 min. The fixed cells were incubated with crystal violet solution (0.1% crystal violet, 20% CH_3_OH) for 1 h. The amount of dye taken up by the monolayer, proportional to the number of viable cells attached to the well bottom, was quantified by extracting the color with 10% acetic acid and measuring the absorbance at a wavelength of 595 nm. The relative viability following treatment with ATRA, LE135 or PLX4032 was used to determine the half maximal inhibitory concentration (IC_50_). By plotting the dose-response curve, the IC_50_ value was estimated as the concentration at the point of 50% cell viability.

### DNA and RNA purification

DNA for quantification of mtDNA and RNA for cDNA synthesis were purified simultaneously with AllPrep DNA/RNA/Protein mini kit (Qiagen) according to the provided protocol.

### Expression analysis

Synthesis of cDNA was performed with the qScript™ XLT cDNA SuperMix (Quanta Bioscience). Gene expression of PGC1α, MITF, RARβ, p14^ARF^, UCP2, ATP5g1, COX5A and NDUFS3 was determined with quantitative real-time PCR on Roche LightCycler 2.0 using the LightCycler FastStart DNA MasterPLUS SYBR Green I kit (Roche). Primers are listed in Supplementary Table 1.

### Immunoblotting

Samples were prepared from cell culture flasks with lysis buffer (SLB) supplemented with colorless β-mercaptoethanol (BPB), Phospho-Stop and protease inhibitor (Thermo Fisher Scientific). Cell lysates were cleared by centrifugation 20,000 rpm for 3 min. Protein concentration was measured using the Qubit Protein Assay Kit (Thermo Fisher Scientific), and 50 μg protein of each sample were loaded to a 10-well SDS, 4-12% Bis-Tris NuPage gel (Invitrogen). Proteins were then separated at 80 V for 30 min, followed by 110 V until completion. Blotting was performed with a semi-dry transfer unit on an ECL nitrocellulose membrane at 3.3 mA/1 cm^2^/1 h/gel. Afterwards the membrane was stained with *Ponceau*. The membrane was blocked in 5% milk for 1 h, then washed twice for 5 min with TBST and stained with anti-AMPK or anti-p-AMPK (Thr172) antibodies (Cell Signaling; 1:2000) in 5% BSA at 4°C and with an anti cyclophilin A antibody (Cell Signaling; 1:5000) as loading control. After three 10-min cycles of washing with TBST, the membrane was stained with the secondary antibody (anti-rabbit; DakoCytomation; 1:2000) for 1 h at room temperature, followed by another 3 wash cycles. Proteins were visualized using ECL Plus Western Blotting Substrate (Thermo Fisher Scientific) 1:1 for 2-3 min.

### Metabolic analysis

Metabolic analysis was performed on melanoma cell lines and melanocytes using a Seahorse XFe96 analyzer (Seahorse Bioscience, Billerica, MA), which performs real-time measurements of the extracellular acidification rate (ECAR) and oxygen consumption rate (OCR). Cells were seeded at 20,000 per well in Seahorse Cell Culture Microplates 24 h prior to running the measurements. Changes in the basal activity and the capacity of the mitochondrial and glycolytic energy systems were determined using the Mito Stress Test Kit and the Glycolysis Stress Test Kit (Agilent Technologies). The assays were performed according to the protocols provided. The Mito Stress Test was performed in the regular culture medium, whereas in the glycolysis stress test, medium was replaced with Seahorse XF Base medium supplemented with L-glutamine (2 mM), pH adjusted to 7.4, 1 h prior to the measurements. For longer exposures (>24 h), cells were treated in culture flasks prior to seeding. All results were normalized to the number of cells seeded, since the concentrations of ATRA and LE135 used did not affect cell growth during 24 h. The protocol for running the assays in the Seahorse machine included cycles of 3 min mixing/3 min measuring. Three independent experiments were performed with 6 replicates of each sample.

### Transfection

The ED-007 melanoma cell line was transfected with pEGFP (control) or pEGFP-p14^ARF^ expression vectors (2 μg vector/2 × 10^6^ cells), both containing GFP as reporter gene. The constructs were obtained as previously described [19]. Transfection was performed using the Amaxa nucleofection technology, buffer V, program T-020, following the protocol recommended by the manufacturer. Successful transfection was verified visually. Stable clones were selected using 400 μg/ml G418 (geneticin; Thermo Fisher Scientific). In the experimental setup, the cells were seeded without G418.

### Quantification of mitochondrial DNA

Mitochondrial DNA was quantified by droplet digital polymerase chain reaction (ddPCR) using the QX200 system (BioRad Laboratories, Hercules, CA, USA). Approximately 0.5 ng DNA was used for each reaction. Mitochondrial copy number was determined by calculating the ratio between a mitochondrial DNA site (mtMinArc) and a single-copy nuclear locus (β2m) as described by Phillips et al. [44]. Primers, probes and experimental conditions are listed in Supplementary Table 2.

### Statistical analysis

Differences between independent data sets were determined with Student's t-test. One-way matched-samples ANOVA was used for statistical analysis of variance between different treatments. Tukey's honest significance difference (HSD) multi-comparison test was used to determine statistical significance.

## SUPPLEMENTARY MATERIALS FIGURES AND TABLES



## Abbreviations

ATRA: all-trans retinoic acid
DCA: dichloroacetate
ECAR: extracellular acidification rate
OCR: oxygen consumption rate
ROS: reactive oxygen species

## References

[1] (2016). Survival Rates for Melanoma Skin Cancer, by Stage.

[2] Nazarian R, Shi H, Wang Q, Kong X, Koya RC, Lee H, Chen Z, Lee MK, Attar N, Sazegar H, Chodon T, Nelson SF, McArthur G (2010). Melanomas acquire resistance to B-RAF (V600E) inhibition by RTK or N-RAS upregulation. Nature.

[3] Johannessen CM, Boehm JS, Kim SY, Thomas SR, Wardwell L, Johnson LA, Emery CM, Stransky N, Cogdill AP, Barretina J, Caponigro G, Hieronymus H, Murray RR (2010). COT drives resistance to RAF inhibition through MAP kinase pathway reactivation. Nature.

[4] Miller AJ, Mihm MC (2006). Melanoma. N Engl J Med.

[5] Abildgaard C, Guldberg P (2015). Molecular drivers of cellular metabolic reprogramming in melanoma. Trends Mol Med.

[6] Ratnikov BI, Scott DA, Osterman AL, Smith JW, Ronai ZA (2017). Metabolic rewiring in melanoma. Oncogene.

[7] Warburg O, Wind F, Negelein E (1927). The metabolism of tumors in the body. J Gen Physiol.

[8] Hall A, Meyle KD, Lange MK, Klima M, Sanderhoff M, Dahl C, Abildgaard C, Thorup K, Moghimi SM, Jensen PB, Bartek J, Guldberg P, Christensen C (2013). Dysfunctional oxidative phosphorylation makes malignant melanoma cells addicted to glycolysis driven by the (V600E) BRAF oncogene. Oncotarget.

[9] Parmenter TJ, Kleinschmidt M, Kinross KM, Bond ST, Li J, Kaadige MR, Rao A, Sheppard KE, Hugo W, Pupo GM, Pearson RB, McGee SL, Long GV (2014). Response of BRAF-mutant melanoma to BRAF inhibition is mediated by a network of transcriptional regulators of glycolysis. Cancer Discov.

[10] McArthur GA, Puzanov I, Amaravadi R, Ribas A, Chapman P, Kim KB, Sosman JA, Lee RJ, Nolop K, Flaherty KT, Callahan J, Hicks RJ (2012). Marked, homogeneous, and early [18F]fluorodeoxyglucose-positron emission tomography responses to vemurafenib in BRAF-mutant advanced melanoma. J Clin Oncol.

[11] Haq R, Shoag J, Andreu-Perez P, Yokoyama S, Edelman H, Rowe GC, Frederick DT, Hurley AD, Nellore A, Kung AL, Wargo JA, Song JS, Fisher DE (2013). Oncogenic BRAF regulates oxidative metabolism via PGC1alpha and MITF. Cancer Cell.

[12] Chapman PB, Hauschild A, Robert C, Haanen JB, Ascierto P, Larkin J, Dummer R, Garbe C, Testori A, Maio M, Hogg D, Lorigan P, Lebbe C (2011). Improved survival with vemurafenib in melanoma with BRAF V600E mutation. N Engl J Med.

[13] Corazao-Rozas P, Guerreschi P, Jendoubi M, Andre F, Jonneaux A, Scalbert C, Garcon G, Malet-Martino M, Balayssac S, Rocchi S, Savina A, Formstecher P, Mortier L (2013). Mitochondrial oxidative stress is the Achille's heel of melanoma cells resistant to Braf-mutant inhibitor. Oncotarget.

[14] Zhang G, Frederick DT, Wu L, Wei Z, Krepler C, Srinivasan S, Chae YC, Xu X, Choi H, Dimwamwa E, Ope O, Shannan B, Basu D (2016). Targeting mitochondrial biogenesis to overcome drug resistance to MAPK inhibitors. J Clin Invest.

[15] Livingstone E, Swann S, Lilla C, Schadendorf D, Roesch A (2015). Combining BRAF(V) (600E) inhibition with modulators of the mitochondrial bioenergy metabolism to overcome drug resistance in metastatic melanoma. Exp Dermatol.

[16] Hoon DS, Spugnardi M, Kuo C, Huang SK, Morton DL, Taback B (2004). Profiling epigenetic inactivation of tumor suppressor genes in tumors and plasma from cutaneous melanoma patients. Oncogene.

[17] Dahl C, Christensen C, Jonsson G, Lorentzen A, Skjodt ML, Borg A, Pawelec G, Guldberg P (2013). Mutual exclusivity analysis of genetic and epigenetic drivers in melanoma identifies a link between p14 ARF and RARbeta signaling. Mol Cancer Res.

[18] Lotan R, Lotan D (1981). Enhancement of melanotic expression in cultured mouse melanoma cells by retinoids. J Cell Physiol.

[19] Christensen C, Bartkova J, Mistrik M, Hall A, Lange MK, Ralfkiaer U, Bartek J, Guldberg P (2014). A short acidic motif in ARF guards against mitochondrial dysfunction and melanoma susceptibility. Nat Commun.

[20] Baldea I, Costin GE, Shellman Y, Kechris K, Olteanu ED, Filip A, Cosgarea MR, Norris DA, Birlea SA (2013). Biphasic pro-melanogenic and pro-apoptotic effects of all-trans-retinoic acid (ATRA) on human melanocytes: time-course study. J Dermatol Sci.

[21] Kawakami T, Ohgushi A, Hirobe T, Soma Y (2017). Analysis of the effects of all-trans retinoic acid on human melanocytes and melanoblasts *in vitro*. J Dermatol.

[22] Vazquez F, Lim JH, Chim H, Bhalla K, Girnun G, Pierce K, Clish CB, Granter SR, Widlund HR, Spiegelman BM, Puigserver P (2013). PGC1alpha expression defines a subset of human melanoma tumors with increased mitochondrial capacity and resistance to oxidative stress. Cancer Cell.

[23] Donadelli M, Dando I, Fiorini C, Palmieri M (2014). UCP2, a mitochondrial protein regulated at multiple levels. Cell Mol Life Sci.

[24] Oberkofler H, Klein K, Felder TK, Krempler F, Patsch W (2006). Role of peroxisome proliferator-activated receptor-gamma coactivator-1alpha in the transcriptional regulation of the human uncoupling protein 2 gene in INS-1E cells. Endocrinology.

[25] Arigony AL, de Oliveira IM, Machado M, Bordin DL, Bergter L, Pra D, Henriques JA (2013). The influence of micronutrients in cell culture: a reflection on viability and genomic stability. Biomed Res Int.

[26] Li Y, Hashimoto Y, Agadir A, Kagechika H, Zhang X (1999). Identification of a novel class of retinoic acid receptor beta-selective retinoid antagonists and their inhibitory effects on AP-1 activity and retinoic acid-induced apoptosis in human breast cancer cells. J Biol Chem.

[27] de The H, Marchio A, Tiollais P, Dejean A (1989). Differential expression and ligand regulation of the retinoic acid receptor alpha and beta genes. EMBO J.

[28] Hardie DG, Hawley SA (2001). AMP-activated protein kinase: the energy charge hypothesis revisited. Bioessays.

[29] Abildgaard C, Dahl C, Basse AL, Ma T, Guldberg P (2014). Bioenergetic modulation with dichloroacetate reduces the growth of melanoma cells and potentiates their response to BRAFV600E inhibition. J Transl Med.

[30] Populo H, Caldas R, Lopes JM, Pardal J, Maximo V, Soares P (2015). Overexpression of pyruvate dehydrogenase kinase supports dichloroacetate as a candidate for cutaneous melanoma therapy. Expert Opin Ther Targets.

[31] Kluza J, Corazao-Rozas P, Touil Y, Jendoubi M, Maire C, Guerreschi P, Jonneaux A, Ballot C, Balayssac S, Valable S, Corroyer-Dulmont A, Bernaudin M, Malet-Martino M (2012). Inactivation of the HIF-1alpha/PDK3 signaling axis drives melanoma toward mitochondrial oxidative metabolism and potentiates the therapeutic activity of pro-oxidants. Cancer Res.

[32] Kaplon J, Zheng L, Meissl K, Chaneton B, Selivanov VA, Mackay G, van der Burg SH, Verdegaal EM, Cascante M, Shlomi T, Gottlieb E, Peeper DS (2013). A key role for mitochondrial gatekeeper pyruvate dehydrogenase in oncogene-induced senescence. Nature.

[33] Murholm M, Isidor MS, Basse AL, Winther S, Sorensen C, Skovgaard-Petersen J, Nielsen MM, Hansen AS, Quistorff B, Hansen JB (2013). Retinoic acid has different effects on UCP1 expression in mouse and human adipocytes. BMC Cell Biol.

[34] Jin W, Xu YP, Yang AH, Xing YQ (2015). *In vitro* induction and differentiation of umbilical cord mesenchymal stem cells into neuron-like cells by all-trans retinoic acid. Int J Ophthalmol.

[35] Tourniaire F, Musinovic H, Gouranton E, Astier J, Marcotorchino J, Arreguin A, Bernot D, Palou A, Bonet ML, Ribot J, Landrier JF (2015). All-trans retinoic acid induces oxidative phosphorylation and mitochondria biogenesis in adipocytes. J Lipid Res.

[36] Tripathy S, Chapman JD, Han CY, Hogarth CA, Arnold SL, Onken J, Kent T, Goodlett DR, Isoherranen N (2016). All-trans-retinoic acid enhances mitochondrial function in models of human liver. Mol Pharmacol.

[37] Watabe H, Soma Y, Ito M, Kawa Y, Mizoguchi M (2002). All-trans retinoic acid induces differentiation and apoptosis of murine melanocyte precursors with induction of the microphthalmia-associated transcription factor. J Invest Dermatol.

[38] De Preter G, Neveu MA, Danhier P, Brisson L, Payen VL, Porporato PE, Jordan BF, Sonveaux P, Gallez B (2016). Inhibition of the pentose phosphate pathway by dichloroacetate unravels a missing link between aerobic glycolysis and cancer cell proliferation. Oncotarget.

[39] Michelakis ED, Webster L, Mackey JR (2008). Dichloroacetate (DCA) as a potential metabolic-targeting therapy for cancer. Br J Cancer.

[40] Corazao-Rozas P, Guerreschi P, Andre F, Gabert PE, Lancel S, Dekiouk S, Fontaine D, Tardivel M, Savina A, Quesnel B, Mortier L, Marchetti P, Kluza J (2016). Mitochondrial oxidative phosphorylation controls cancer cell's life and death decisions upon exposure to MAPK inhibitors. Oncotarget.

[41] Bauer D, Werth F, Nguyen HA, Kiecker F, Eberle J (2017). Critical role of reactive oxygen species (ROS) for synergistic enhancement of apoptosis by vemurafenib and the potassium channel inhibitor TRAM-34 in melanoma cells. Cell Death Dis.

[42] Chen MC, Hsu SL, Lin H, Yang TY (2014). Retinoic acid and cancer treatment. Biomedicine (Taipei).

[43] Robinson J, Roberts CH, Dodi IA, Madrigal JA, Pawelec G, Wedel L, Marsh SG (2009). The European searchable tumour line database. Cancer Immunol Immunother.

[44] Phillips NR, Sprouse ML, Roby RK (2014). Simultaneous quantification of mitochondrial DNA copy number and deletion ratio: a multiplex real-time PCR assay. Sci Rep.

