# Supplementary material for: Inhibition of retinoic acid receptor β signaling confers glycolytic dependence and sensitization to dichloroacetate in melanoma cells
Source: Oncotarget. 2017 Aug 24;8(48):84210–23. doi: 10.18632/oncotarget.20476 (PMC5663589; doi:10.18632/oncotarget.20476)
Supplement: Supplementary file 1 [file oncotarget-08-84210-s001.pdf]

Inhibition of retinoic acid receptor β signaling confers glycolytic dependence and sensitization to dichloroacetate in melanoma cells

SUPPLEMENTARY MATERIALS

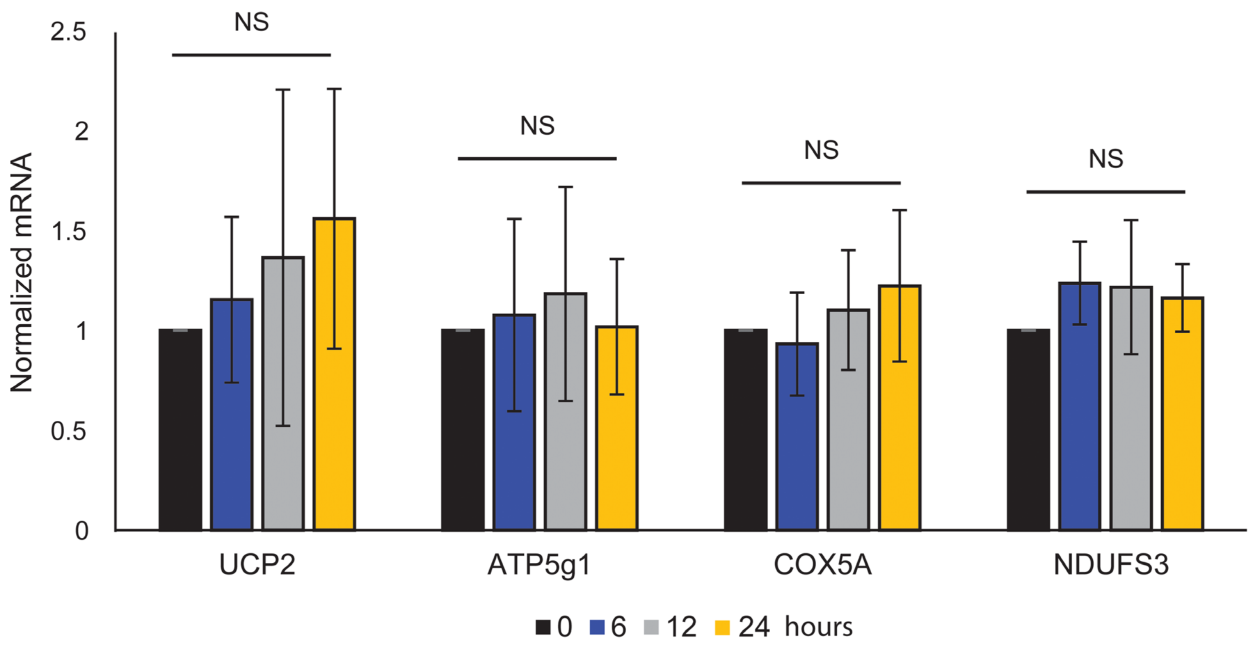

Supplementary Figure 1: Relative mRNA expression of UCP2 and three markers of mitochondrial activity (ATP5g1, COX5A, NDUFS3) in melanocytes treated with ATRA (0.1 µM) for 6, 12 and 24 h, normalized to RPLP0 expression. Data represent the average values ± standard deviation of four biological replicates. Statistical comparison with the untreated control showed no statistical significance (NS).

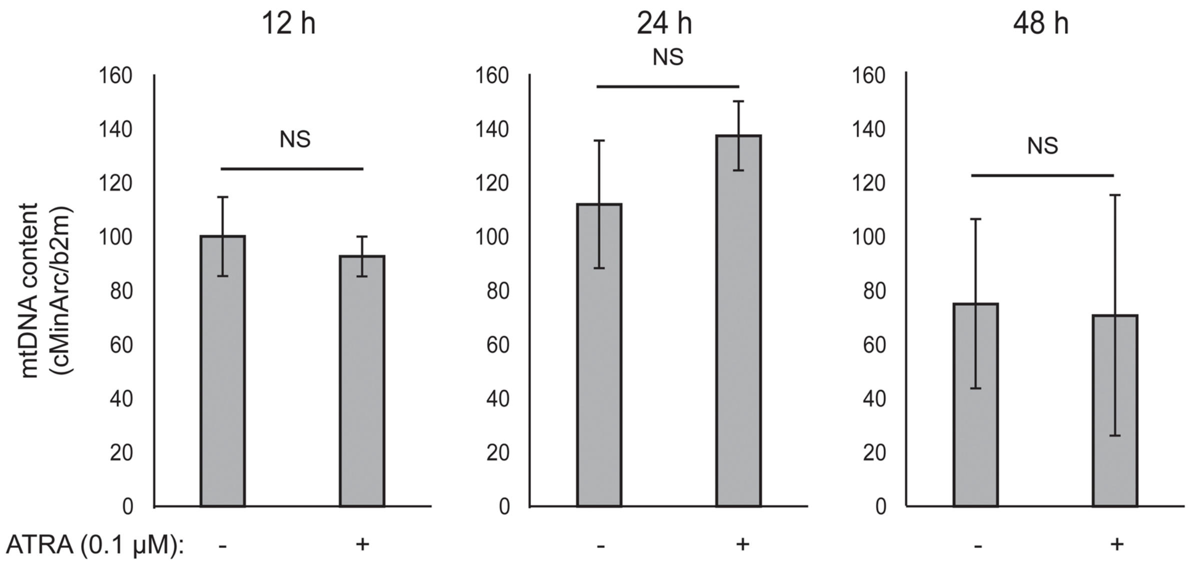

Supplementary Figure 2: Mitochondrial DNA (mtDNA) content after 12, 24 and 48 h of treatment with ATRA (0.1 µM). The diagrams for 12 and 24 h represent the average values ± standard deviation of 3 measurements. The data after 48 h are the average of 3 biological replicates. Statistical comparison with the untreated control showed no statistical significance (NS).

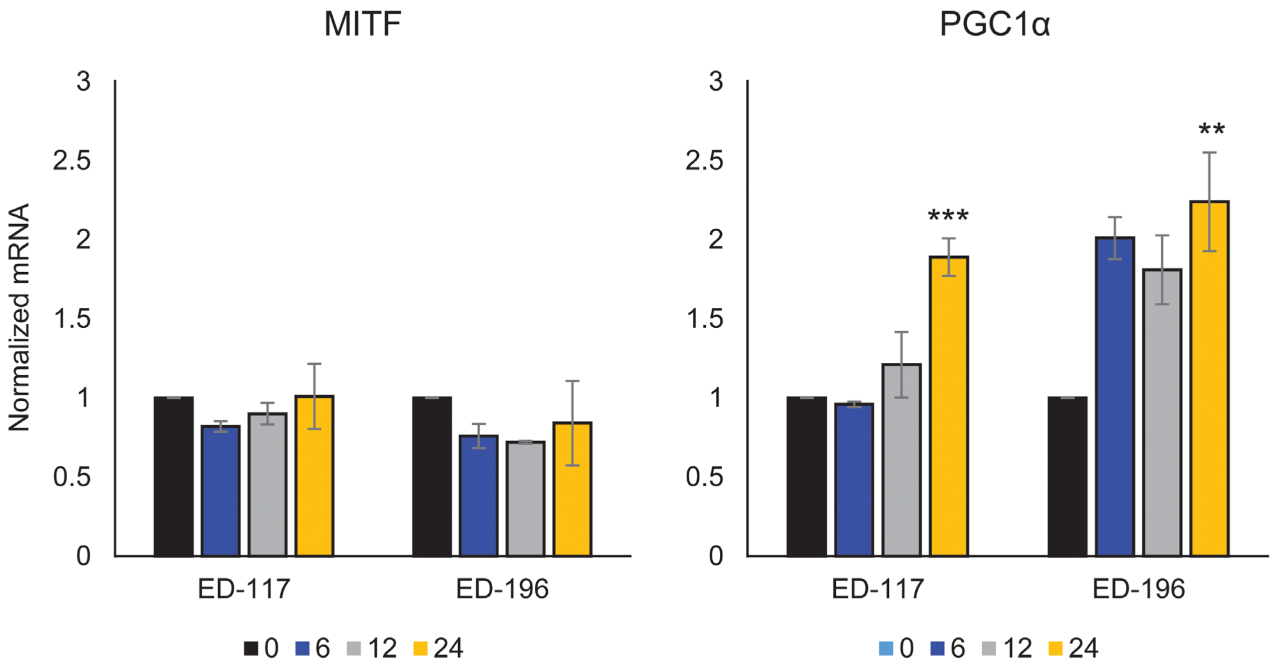

Supplementary Figure 3: Relative mRNA expression of MITF and PGC1a in ED-117 and ED-196 cells treated with ATRA (0.1 µM) for 6, 12 and 24 h, normalized to RPLP0 expression. Data represent the average values of 3 measurements ± standard deviation.

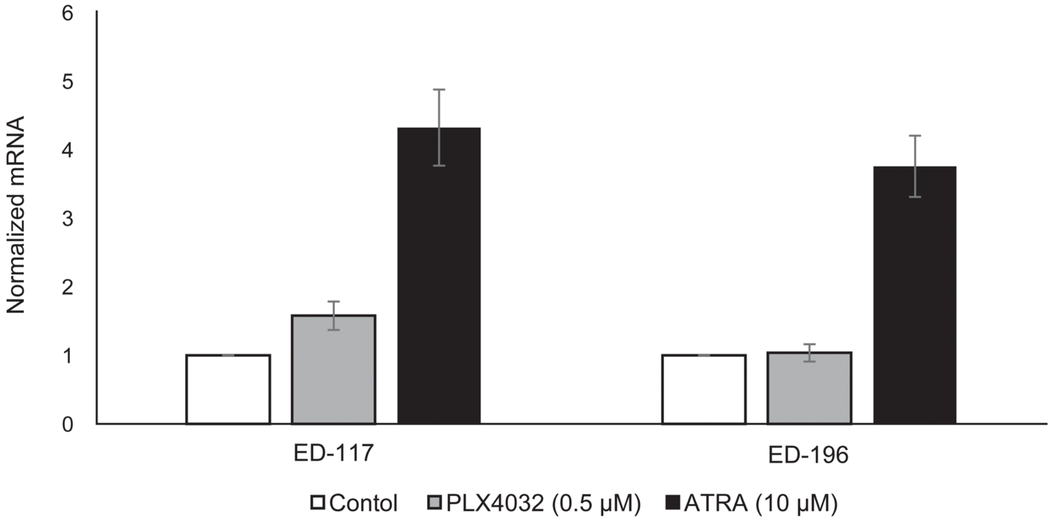

Supplementary Figure 4: Relative expression of RARβ following treatment with PLX4032 (0.5 µM) and ATRA (10 µM) for 24 h, normalized to RPLP0 expression. Data represent the average values of 3 measurements ± standard deviation.

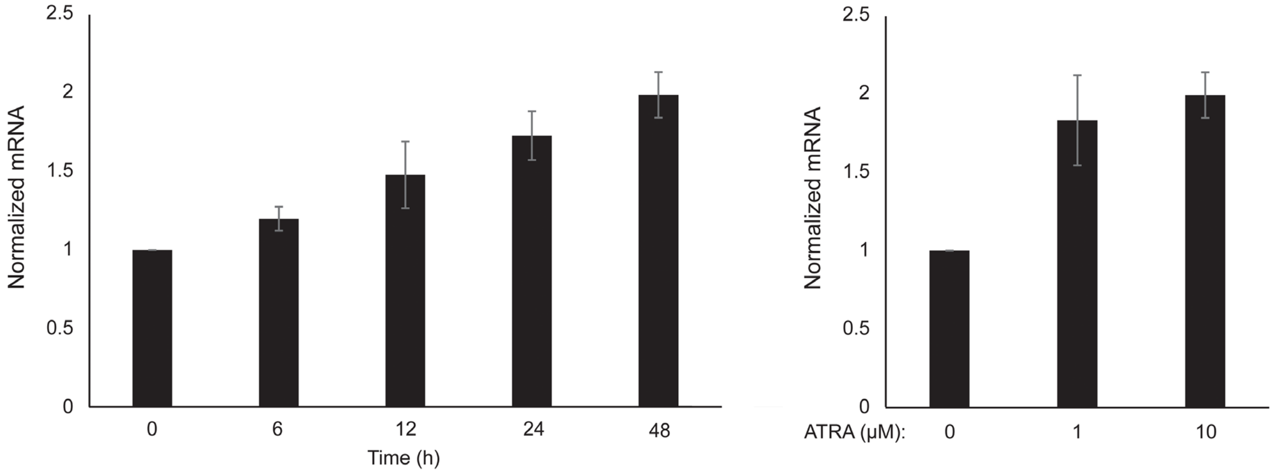

Supplementary Figure 5: Relative expression of p14^{ARF} in ED-117 following treatment with ATRA (10 µM) for 6-48 h (left panel) and ATRA (1-10 µM) for 48 h (right panel), normalized to RPLP0 expression. Data represent the average values of 3 measurements ± standard deviation.

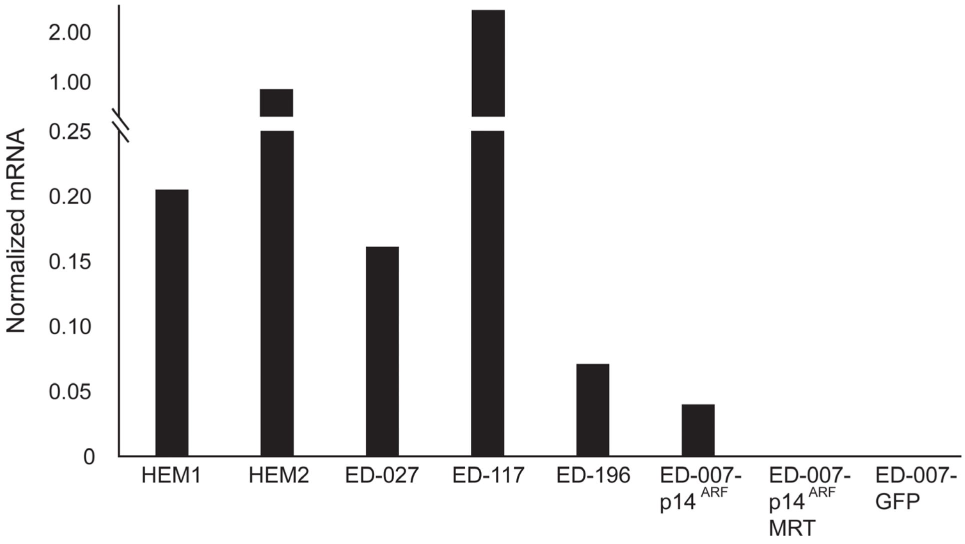

Supplementary Figure 6: Relative expression of p14^{ARF} in two different batches of human epidermal melanocytes (HEM1 and HEM2), ED-027, ED-117, ED-196, and ED-007-p14^{ARF}. ED-007-p14^{ARF} cells express p14^{ARF} at a low level compared to HEM, but comparable to some of the other melanoma cell lines. MRT, minus reverse transcriptase control.

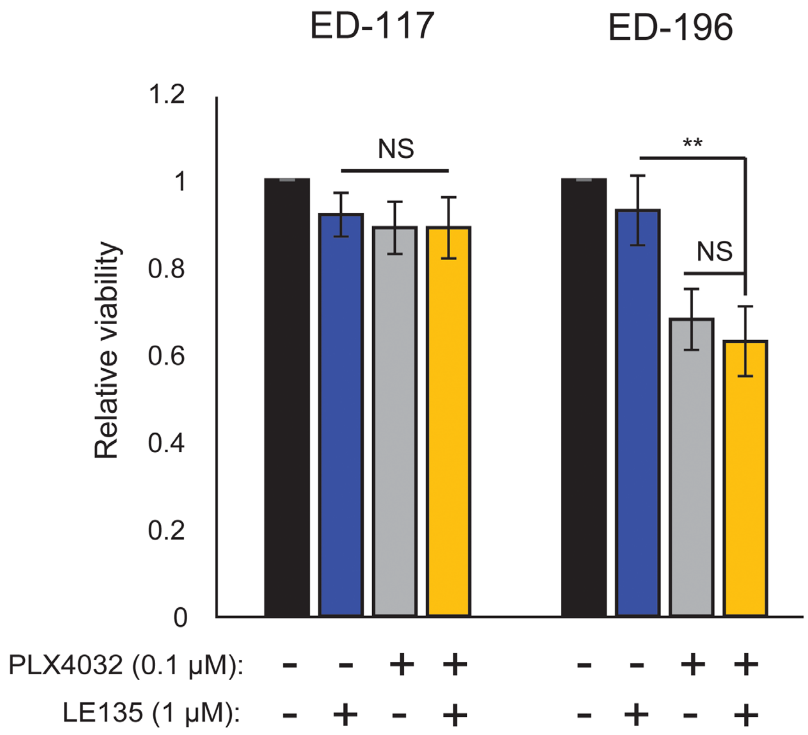

Supplementary Figure 7: Relative viability of BRAF^{V600E}–mutated melanoma cell lines (ED-117 and ED-196) treated with LE135 (1 μM), PLX4032 (0.1 μM), the combination or vehicle control for 6 days. Data represent the average of ≥3 independent experiments ± standard deviation.

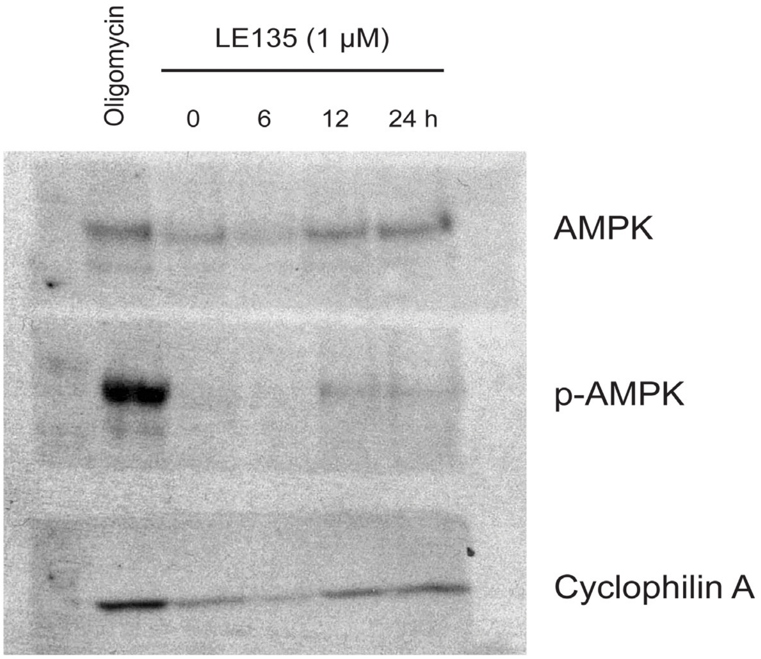

Supplementary Figure 8: Immunoblotting of AMPK and p-AMPK in melanocytes after treatment with LE135 for 6-24 h. Melanocytes treated with oligomycin A (10 μM) for 4 h were used as a positive control for AMPK activation; cyclophilin A was used as loading control.

Supplementary Table 1: Primer sequences for qPCR

Gene	Forward primer	Reverse primer
PGC1α*	GTAAATCTGCGGGATGATGG	AATTGCTTGCGTCCACAAA
MITF**	CCGTCTCTCACTGGATTGGT	TACTTGGTGGGGTTTTCGAG
$p14^{ARF***}$	CCCTCGTGCTGATGCTACTGA	CATGACCTGGTCTTCTAGGAAGC
RARβ***	TCCTGGATTTCTACACTGCG	AAGCAGGGTTTGTACACTCG
UCP2	AAGACCATTGCCCGAGAGG	TTGGCTTTCAGGAGGGCAT
ATP5g1*	ATCATTGGCTATGCCAGGAA	ATGGCGAAGAGGATGAGGA
COX5A*	GGGAATTGCGTAAAGGGATAA	TCCTGCTTTGTCCTTAACAACC
NDUFS3*	GCTGACGCCCATTGAGTCTG	GGAACTCTTGGGCCAACTCC
RPLP0	ACTAAAATCTCCAGGGGCACC	ATGACCAGCCCAAAGGAGAA

*Primer sequences published by Vazquez et al. 2013 [22].
**Primer sequences published by Haq et al. 2014 [11].
***Primer sequences published by Dahl et al. 2013 [17].

Supplementary Table 2: Experimental conditions for quantification of mtDNA with ddPCR

	mtMinArc	β2m
Forward primer*	CTAAATAGCCCACACGTTCCC	GCTGGGTAGCTCTAAACAATGTATTCA
Reverse primer*	AGAGCTCCCGTGAGTGGTTA	CCATGTACTAACAAATGTCTAAAATGGT
Probe*	6FAM-CATCACGATGGATCACAGGT(NFQ)	VIC-CAGCAGCCTATTCTGC(NFQ)
Primer conc.	75 nM	500 nM
Annealing temp.	50°C	52°C
No. of cycles	40	40

*Primer and probe sequences published by Phillips et al. [44].
